# A case report of Kaposiform haemangioendothelioma; response with propranolol and steroids

**DOI:** 10.1186/s13569-020-00134-8

**Published:** 2020-07-30

**Authors:** Saurav Verma, Ekta Dhamija, Adarsh Barwad, Venkatesan S. Kumar, Sameer Rastogi

**Affiliations:** 1 Department of Medical Oncology, Dr. B.R.A. Institute Rotary Cancer Hospital, All India Institute of Medical Sciences, New Delhi, India; 2 Department of Radiodiagnosis, Dr. B.R.A Institute Rotary Cancer Hospital, All India Institute of Medical Sciences, New Delhi, India; 3 Department of Pathology, All India Institute of Medical Sciences, New Delhi, India; 4 Department of Orthopaedics, All India Institute of Medical Sciences, New Delhi, India; 5 Sarcoma Medical Oncology Clinic, Department of Medical Oncology, All India Institute of Medical Sciences, New Delhi, India

**Keywords:** Kaposiform hemangioendothelioma, Propranolol, Steroids

## Abstract

**Background:**

Kaposiform haemangioendothelioma is a rare vascular tumor and may involve skin, deep soft tissue or bone. It is a locally aggressive tumor usually seen in infants. Here we report a case of kaposiform hemagioendothelioma in a child who responded to propranolol and steroids.

**Case presentation:**

A 3-year-old male child presented with a swelling below his right knee with characteristic violet skin lesion. There was no evidence of Kasabach–Merritt phenomenon. After no improvement with several attempts at debridement and anti-tubercular treatment; a diagnosis of Kaposiform Haemangioendothelioma was reached on the basis of overall clinical picture and histology. The child was treated with propranolol and steroids and had an excellent clinical response and a near complete resolution on imaging at 5 months.

**Conclusions:**

These cases are often misdiagnosed and despite a delay in diagnosis have good outcomes with appropriate multimodality management. This case highlights the unique and typical characteristics of kaposiform haemangioendothelioma.

## Background

Kaposiform haemangioendothelioma (KH), first described by Zuckerberg et al. in 1993, is a rare vascular tumor and may involve skin, deep soft tissue or bone [1]. It is usually seen in infants and children with a median age at diagnosis of 6.5 months (range 0.3–14.0 months) and an incidence of less than 1 per million [2, 3]. Although extremely seldom, it can also occur in older children and Adolescents and Young Adults (AYAs). It is a locally aggressive tumor with no report of distant metastasis [4]. It may involve extremities, torso (including retroperitoneum and intrathoracic cavity) or cervicofacial region in decreasing order of frequency. The term “kaposiform” relates to its resemblance to Kaposi’s sarcoma, with compact spindled tumor cells, displaying slit like vessels and expressing CD34, usually lacking factor VIII-AG, and surrounded by a population of factor XIIIA-positive cells. The term “haemangioendothelioma” implies dilemma regarding its biologic behaviour, a tumor with intermediate malignancy situated somewhere between the spectrum of haemangioma and angiosarcoma [1]. The diagnosis is made on the basis of clinical presentation, imaging and histopathologic examination along with adequate immunohistochemistry. The mainstay of treatment remains complete surgical removal with wide margins. When tumor is unresectable or surgery is associated with unacceptable morbidity other treatment modalities like angiography followed by embolization, radiotherapy, vincristine chemotherapy, pharmacotherapy with propranolol, steroids or sirolimus have been used with varying rates of success [5–9]. Some patients may develop a life-threatening complication known as Kasabach–Merritt phenomenon (KMP). KMP is thrombocytopenia (average platelet count 25,000/mm^3^; range 3000–60,000/mm^3^) with hypofibrinogenemia resulting from intralesional platelet activation, trapping, and consumption [10]. The natural history and long-term outcomes after treatment are poorly understood. Here we report a case treated with propranolol and steroids.

## Case presentation

**Fig. 1:**
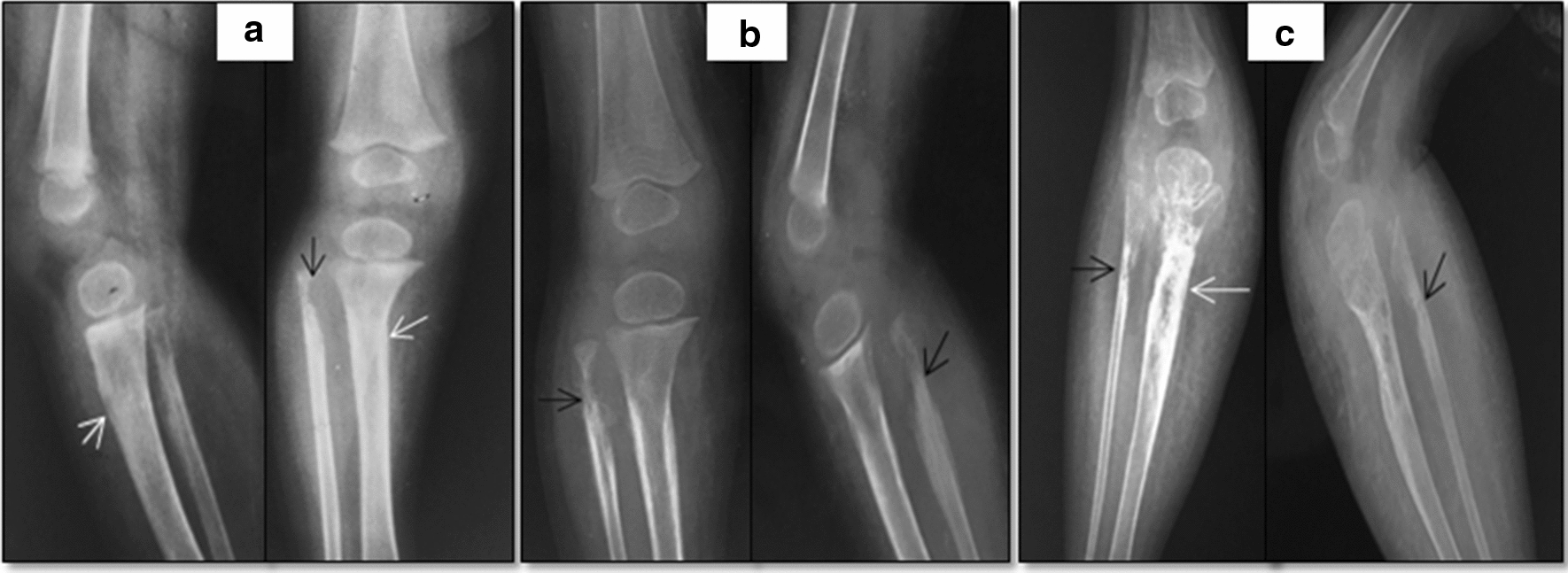
Sequential radiographs showing soft tissue and osseous changes in tibia (white arrows) and fibula (black arrows) over span of two years. Initial X-ray of 2016 **a** shows ill-defined sclerosis in proximal tibia and cortical erosion along the medial cortex of fibula which further progresses to circumferential cortical erosion in follow up radiograph of 2017 (**b**). The X-ray following three surgical debridements **c** demonstrates mixed lytic sclerotic changes involving proximal tibia and fibula. Gradually progressive soft tissue swelling is seen with predominant fat proliferation involving calf of right leg

A 3-year-8-month old male presented with a violet skin lesion and a swelling below his right knee which was first noticed at an age of 7 months. The swelling gradually increased in size over the next two years and was associated with pain, low grade intermittent fever and decreased appetite. At this time a radiograph was obtained which revealed lytic areas involving head and proximal shaft of right tibia and fibula (images not shown). An exploration of lesion was done in December, 2015 and histopathology revealed fibrofatty tissue with evidence of chronic inflammation. A provisional diagnosis of osteomyelitis was made and treatment with broad spectrum antibiotics was initiated. However, there was no symptomatic relief and the swelling progressed in size. After a repeat imaging, 2nd debridement was done in June, 2016 (Fig. 1a) and antibiotics were continued. Histopathology on this occasion showed normal bone with few areas of necrosis and no evidence of granuloma or malignancy. Despite surgery, the symptoms persisted and 3rd debridement was done in December, 2016. Results were again disappointing and he was started on empirical ATT from April, 2017 (Fig. 1b).

**Fig. 2:**
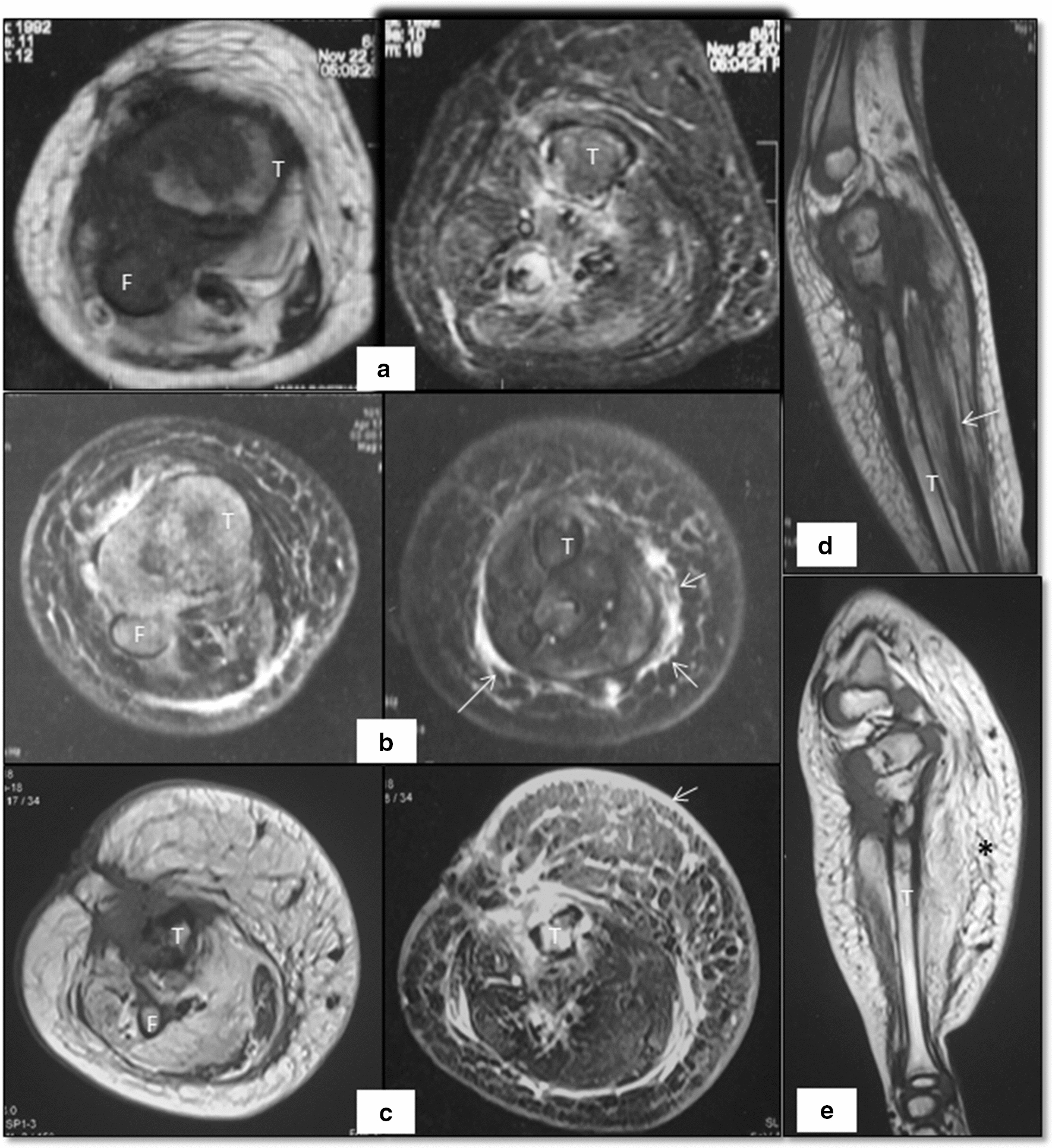
Sequential MRI of patient with Kaposiform haemangioendothelioma. Initial axial MR images **a** of 2016 show cortical destruction, marrow oedema involving tibia (T) and fibula (F) with surrounding soft tissue oedema on T1 (left) as well as STIR (right) sequences. Subsequently performed MRI in 2017 reveals increase in calf diameter and marrow oedema in both bones with visualization of plaque like hyperintensity along the muscle plane on STIR images (arrows in **b**). The recent MR images demonstrate trans-compartment involvement of skin (arrow in **c**) as thickening and hyperintense signal intensity; subcutaneous plane as fat proliferation which shows reticular pattern/lymphedema; muscles as atrophy and fat proliferation within the intermuscular plane and bones with marrow signal alteration. The similar changes are also evident on T1 weighted sagittal images of 2016 (arrow in **d**) and 2018 (asterisk in **e**)

**Fig. 3:**
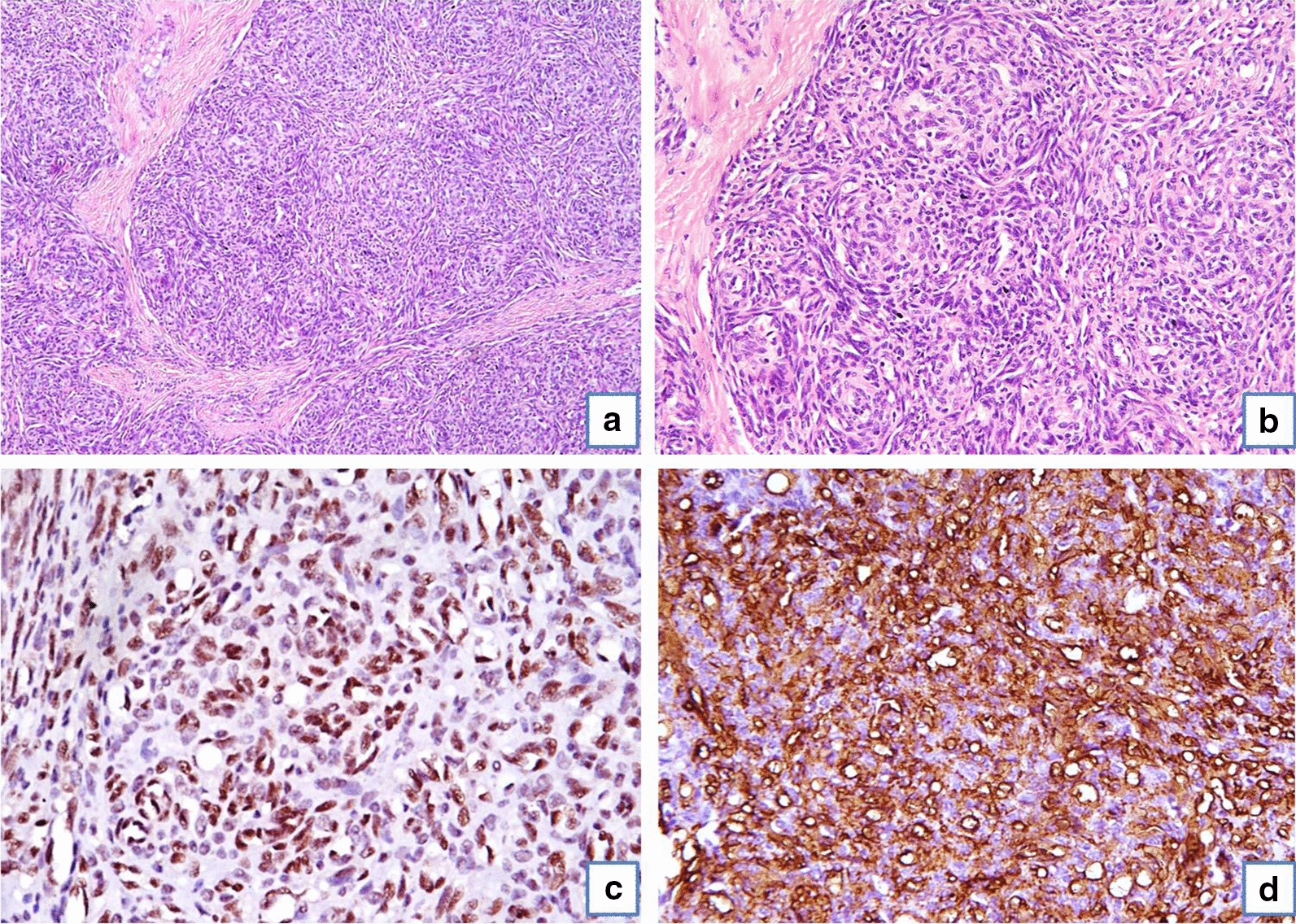
Histopathology of Kaposiform haemangioendothelioma. **a** Low power photomicrograph of the tumor showing nodular architecture with spindle cells arranged in cannon ball fashion with slit like ill formed vascular channels. (H&E 100×). **b** High power photomicrograph showing cells exhibiting mild pleomorphism with elongated nuclei, finely dispersed chromatin and scanty to moderate cytoplasm. Few of the vascular spaces showed RBC. There were occasional mitotic figures. (H&E 200×). **c** Immunostaining for CD31 showing diffuse cytoplasmic positivity in tumor cells. **d** Immunostaining for FLI-1 showing nuclear positivity in tumor cells

He presented to our hospital in August, 2017. The child had a swelling below knee over the right leg which was hard in consistency. The skin over the tumor was of violet color with increased temperature. The child had a poor nutritional status. His complete blood counts were suggestive of anaemia, leucocytosis and normal platelet count. Liver and kidney function tests were normal. Peripheral smear revealed microcytic and hypochromic RBCs. Bone marrow studies were normal. Workup for primary immunodeficiency, bacterial/fungal infection and tuberculosis was negative. A radiograph of right leg showed lytic sclerotic destructive lesion with midzone of transition seen involving right proximal tibia and fibula with soft tissue component (Fig. 1c). MRI done in 2016 and 2017 (Fig. 2) revealed destruction of the right proximal tibia and fibula with marrow oedema, oedematous changes in surrounding soft tissue, atrophy of the calf muscles and a sinus tract due to prior surgical interventions. A bone biopsy was done which showed fibro-collagenous tissue with mild chronic inflammation. He was considered to have chronic tubercular osteomyelitis and continued on ATT till August, 2018 with no response. A repeat biopsy of the lesion in August, 2018 showed a tumor with nodular architecture. Immunohistochemistry was positive for CD34, FLI-1 and CD31 (Fig. 3). It was negative for EMA, desmin and myogenin. Skin biopsy showed features of acanthotic epidermis with dense fibrosis in the dermis and similar tumor in deep dermis. Bone biopsy had infiltration of bone by similar tissue. Fat biopsy showed fibroadipose tissue with vascular proliferation. A diagnosis of KH was made and he was referred to our sarcoma clinic in September, 2018. He was started on treatment with propranolol at a dose of 0.6 mg/kg twice a day for 1 week followed by 1.1 mg/kg for 2 weeks and 1.7 mg/kg thereafter along with prednisone at 2 mg/kg. On follow-up after a month child was better; with a slight decrease in size of swelling and pain and was able to sleep without analgesics. His appetite improved. On follow up at 3 month he had an excellent clinical response. Imaging with MRI at 5 months revealed a near complete resolution. He developed cushingoid features on steroids which resolved on tapering of steroids.

The treatment plan for surgery was discussed with parents. However, parents have preferred to defer any extensive surgery and decided to continue with medical management.

## Discussion and conclusions

KH is set apart from other common and benign vascular lesions of childhood like juvenile haemangioma, cavernous haemangioma, angiomatosis and endovascular papillary angio-endothelioma by its unique histopathological features and locally invasive nature [1].

The most common clinical presentation of KH is a characteristic violet cutaneous lesion which infiltrates the underlying skin on the extremities [4]. Our patient presented with typical clinical features of a cutaneous lesion and a swelling in the extremity in infancy. There was no evidence of KMP, profound thrombocytopenia resulting from intralesional platelet trapping, in our patient. In a study by Croteau et al. 71% patients of KH had evidence of KMP at diagnosis [3]. Studies have indicated that truncal involvement and larger size may predict for more serious involvement including KMP [11, 12].

Due to rarity of KH, there are no prospective studies and only case series and case reports are available to guide management. For resectable lesions, surgery is the mainstay of management as there is no spontaneous regression in KH. For non-resectable lesions or for those in which surgery will entail significant morbidity pharmacological management is desirable to shrink tumor size. Such pharmacological treatment is also used in patients presenting with KMP to correct coagulopathy and decrease tumor size. Trans-arterial embolization has been used to treat KH of scalp and cervical region [5, 13]. Corticosteroids have been widely used as a first line agent in most cases with questionable benefit as a sole agent. Propranolol has been used in vascular tumours like angiosarcoma as part of combination metronomic chemotherapy with clinically significant responses [7, 14]. Propranolol with and without steroids has shown variable responses with some cases having long lasting remissions in KH patients [8, 15]. Vincristine has been used as a sole agent and with steroids especially in patients with KMP with improvement in platelet counts and decrease in tumor size in some patients [6, 16]. VAT (Vincistine-Aspirin-Ticlopidine) combination therapy also showed clinical response [17, 18]. In critically ill patients chemotherapy combinations utilising cyclophosphamide, methotrexate, vincristine and actinomycin-D have been utilised [19]. Sirolimus, a mammalian target of Rapamycin (mTOR) inhibitor, has been demonstrated to have anti-angiogenic activity in pre-clinical models. In some patients sirolimus at a dose of (0.1 mg/kg/day) resulted in rapid and dramatic response [9, 20]. Platelet transfusion is only indicated for active bleeding and/or immediately prior to surgery as it may exacerbate KMP [21].

In 2013, Drolet et al. presented consensus guidelines based on limited clinical literature [22]. Initial diagnostic workup should consist of CBC with platelet count, coagulation studies (including PT/PTT/fibrinogen and D-Dimer), local imaging with MRI with contrast and tissue biopsy. For cases of KH associated with KMP, first-line therapy with intravenous vincristine 0.05 mg/kg once weekly AND oral prednisolone 2 mg/kg/d OR intravenous methylprednisolone 1.6 mg/kg/d was recommended. For cases of KH that require intervention because of growth or symptoms but do not have KMP, oral prednisolone 2 mg/kg/d was recommended as the first-line therapy. Treatment with aspirin at an antiplatelet dose of 2–5 mg/kg/d could be considered as adjunctive therapy.

**Fig. 4:**
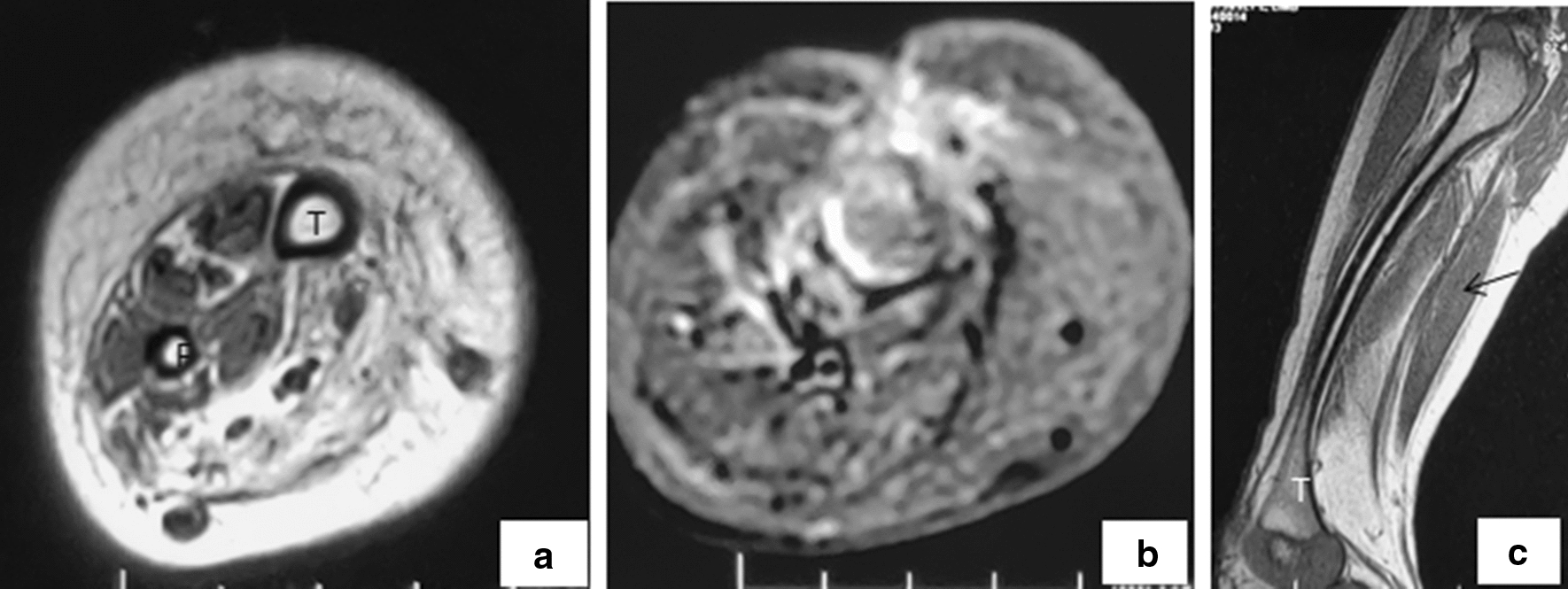
Response to treatment on MRI. Follow up MRI shows resolution of reticular hyperintense pattern on T1 (**a**) and STIR (**b**) axial images with increase in bulk of the calf muscles (**c**) as compared to pre-treatment scan (Fig. 2)

In our patient we started with propranolol and steroids with a clinical response at 1 month and an excellent response on imaging at 5 months of treatment (Fig. 4). The long term adverse effects of steroid treatment, including growth retardation, a cushingoid appearance, and opportunistic infections have been reported [23]. In the above cases steroids were gradually tapered following development of cushingoid facies.

There can be a delay in diagnosis of such rare tumours, as in our patient. Since KH is a rare tumor, the delay may be due to lack of expert sarcoma pathologist and dedicated sarcoma oncologists in India [24]. More collaboration between different specialists like surgery, orthopaedics, radiology and pathology is required for streamlining and optimizing patient management. A simple way is to develop multidisciplinary tumor board at tertiary care institutions and dedicated sarcoma clinics.

## Abbreviations

KH: Kaposiform Haemangioendothelioma
AYAs: Adolescents and Young Adults
CD: Cluster of differentiation
KMP: Kasabach–Merritt phenomenon
ATT: Anti-tubercular treatment
RBCs: Red blood cells
MRI: Magnetic resonance imaging
FLI1: Friend leukemia integration 1 transcription factor
EMA: Epithelial membrane antigen
VAT: Vincistine–Aspirin–Ticlopidine
mTOR: Mammalian target of rapamycin
PT: Prothrombin time
PTT: Partial thromboplastin time
